# Antibodies in the Diagnosis of Coeliac Disease: A Biopsy-Controlled, International, Multicentre Study of 376 Children with Coeliac Disease and 695 Controls

**DOI:** 10.1371/journal.pone.0097853

**Published:** 2014-05-15

**Authors:** Johannes Wolf, Dirk Hasenclever, David Petroff, Thomas Richter, Holm H. Uhlig, Martin W. Laaβ, Almuthe Hauer, Martin Stern, Xavier Bossuyt, Jan de Laffolie, Gunter Flemming, Danilo Villalta, Wolfgang Schlumberger, Thomas Mothes

**Affiliations:** 1 Institute of Laboratory Medicine, Clinical Chemistry and Molecular Diagnostics, Medical Faculty of the University and University Hospital, Leipzig, Germany; 2 Institute for Medical Informatics, Statistics & Epidemiology of the University, Leipzig, Leipzig, Germany; 3 Coordination Centre for Clinical Trials of the University, Leipzig, Germany; 4 Children's Hospital of the Clinical Centre “Sankt Georg”, Leipzig, Germany; 5 Translational Gastroenterology Unit, Experimental Medicine, University of Oxford, John Radcliffe Hospital, Oxford, England; 6 University Children's Hospital, Dresden, Germany; 7 University Children's Hospital, Graz, Austria; 8 University Children's Hospital, Tübingen, Germany; 9 Laboratory Medicine, Immunology, University Hospitals Leuven, Catholic University, Leuven, Belgium; 10 University Children's Hospital, Gieβen, Germany; 11 University Children's Hospital, Leipzig, Germany; 12 Allergy and Clinical Immunology Unit, Azienda Ospedaliera “San Maria degli Angeli”, Pordenone, Italy; 13 EUROIMMUN AG Labormedizinische Diagnostika AG, Lübeck, Germany; Tulane University, United States of America

## Abstract

Diagnosis of coeliac disease (CD) relies on a combination of clinical, genetic, serological and duodenal morphological findings. The ESPGHAN suggested that biopsy may not be necessary in all cases. New guidelines include omission of biopsy if the concentration of CD-specific antibodies exceeds 10 times the upper limit of normal (10 ULN) and other criteria are met. We analysed the 10 ULN criterion and investigated multiple antibody-assays. Serum was collected from 1071 children with duodenal biopsy (376 CD patients, 695 disease-controls). IgA-antibodies to tissue transglutaminase (IgA-aTTG), IgG-antibodies to deamidated gliadin peptides (IgG-aDGL) and IgA-endomysium antibodies (IgA-EMA) were measured centrally. We considered 3 outcomes for antibody test procedures utilizing IgA-aTTG and/or IgG-aDGL: positive (≥10 ULN, recommend gluten-free diet), negative (<1 ULN, no gluten-free diet) or unclear (perform biopsy). Positive (PPV) and negative (NPV) predictive values were based on clear test results. We required that they and their lower confidence bounds (LCB) be simultaneously very high (LCB >90% and PPV/NPV >95%). These stringent conditions were met for appropriate antibody-procedures over a prevalence range of 9–57%. By combining IgG-aDGL with IgA-aTTG, one could do without assaying total IgA. The PPV of IgG-aDGL was estimated to be extremely high, although more studies are necessary to narrow down the LCB. The proportion of patients requiring a biopsy was <11%. The procedures were either equivalent or even better in children <2 years compared to older children. All 310 of the IgA-aTTG positive children were also IgA-EMA positive. Antibody-assays could render biopsies unnecessary in most children, if experienced paediatric gastroenterologists evaluate the case. This suggestion only applies to the kits used here and should be verified for other available assays. Confirming IgA-aTTG positivity (≥10 ULN) by EMA-testing is unnecessary if performed on the same blood sample. Prospective studies are needed.

## Introduction

Coeliac disease (CD) is an autoimmune mediated enteropathy with tissue transglutaminase (TTG) as autoantigen and is triggered by an abnormal immune response to wheat gluten and related cereal peptides in genetically predisposed persons. The clinical presentation ranges from typical malabsorption signs to rather atypical symptoms and conditions that can affect any organ system.

Until recently, the diagnosis of CD was based on the assessment of the highly variable clinical status, assays of different specific antibodies, the histological evaluation of intestinal biopsies, and the response to gluten-free diet [1]. The new guidelines of the European Society of Paediatric Gastroenterology, Hepatology and Nutrition (ESPGHAN) reflect the changing role of antibodies in the diagnosis of CD. In the past, an elevated antibody concentration was regarded as the main reason for a subsequent biopsy. The new guidelines [2] define CD as a variable combination of gluten-dependent clinical manifestations, of concentrations of CD-specific antibodies, of HLA-DQ2 or HLA-DQ8 haplotypes, and of enteropathy. Thus, antibodies are now already included in the definition. The new guidelines also raised the question of providing a diagnosis without duodenal biopsies. In children and adolescents with typical signs or symptoms suggestive of CD, a concentration above ten times the upper limit of normal (10 ULN) of IgA antibodies against TTG (aTTG) was considered an important precondition for this. Confirmation of antibody positivity by IgA-endomysium antibodies (EMA), HLA-DQ2 or HLA-DQ8 in further blood samples and response to a gluten-free diet complete the diagnosis. Moreover, various groups have suggested testing the performance of assays measuring antibodies against deamidated gliadin peptides (aDGL) [2], [3].

The 10 ULN suggestion was derived from 3 studies, all applying the same antibody test. The first study found only CD patients among 91 biopsied adults with IgA-aTTG ≥10 ULN but none of the 7 control patients [4]. The second study identified Marsh 3 lesions in 78 of 79 patients (adults and children) with IgA-aTTG ≥10 ULN [5]. The third study stated that strongly positive tTG antibody titres were sufficient for CD diagnosis in 97 children, but controls were not included [6]. The guidelines conclude that the new recommendations in clinical practice should be evaluated prospectively.

The performance of antibody tests can be assessed by estimation of their positive predictive values (PPV, proportion of CD patients amongst positively tested individuals) and their negative predictive values (NPV, proportion of CD patients amongst individuals tested to be negative).

PPV and NPV depend strongly on prevalence (pre-test probability). The studies cited above were based on very high prevalence of up to 100%. However, pre-test probability in symptomatic patients in clinical practice may be as low as 3 to 10% [7]–[9]. Studies on the performance of antibody assays at higher cut-offs in the diagnosis of CD in children are still rare.

Here, we report on antibody data from 1071 children, who underwent endoscopy due to gastrointestinal complaints. We investigate diagnostic procedures based on IgA-aTTG measurements alone and in combination with IgG-aDGL. We chose this second antibody because it is directed against an antigen different from TTG and since it may detect antibodies in the case of IgA deficiency. In particular we look at diagnostic procedures with three possible test outcomes: clearly negative, clearly positive (≥10 ULN) and indeterminate. The idea is that clear cases can be spared biopsy while indeterminate cases form a grey zone and require additional diagnostic information (e.g. biopsy). We determine ranges of prevalence for which these antibody based diagnostic procedures are reliable. Our predictions are to be prospectively confirmed in an ongoing antibody trial, AbCD [10].

## Materials and Methods

### Ethics Statement

The study was approved by the ethical committees of the University of Leipzig, the Medical Faculty of the Technical University Carl Gustav Carus Dresden, the Medical University Graz, the Medical Faculty of the Eberhard Karls University and the University Hospital Tübingen, the Medical Faculty of the Ludwig Maximilians University of Munich, the Medical Faculty of the Justus Liebig University Hospital Giessen, the Universitaire Ziekenhuizen Kuleuven, and the Azienda Ospedaliera di Padova. Informed consent was not given by all participants and/or next of kin/caregiver. If there was no informed consent, patient records/information was anonymized and de-identified prior to analysis.

### Patients

We analysed serum from 1071 children consisting of 376 CD patients and 695 control patients (477 boys and 594 girls, mean age 8.3 years, range 0.3–17.9 years, biopsied between 1998 and 2013) in whom CD was excluded by means of duodenal biopsy. A flowchart describing how we arrived at these 1071 children can be found in Figure S1. Data from 627 patients were already included in recent publications [11]–[14] but data of 444 patients are reported for the first time. The patients were recruited from ten European centres.

Selective IgA deficiency (sIgAD) was found in three control patients and 24 CD patients, one of whom had common variable immunodeficiency (CVID). There were 23 children with type 1 diabetes mellitus (20 CD patients and three control patients). The control group also comprised 81 patients with inflammatory bowel disease.

Patients were diagnosed and antibodies were tested as described [11]–[14]. In brief, serum was collected around the time of the diagnostic duodenal biopsies. All patients were biopsied while on a normal diet due to suspicion of CD or other gastrointestinal disorders. For the CD patients, the Marsh-Oberhuber classification of the small bowel biopsies was: Marsh 1, n = 3; Marsh 2, n = 8; Marsh 3, n = 344 (Marsh 3A, n = 69; Marsh 3B n = 115; Marsh 3C, n = 151; not sub-classified, n = 9). One CD patient showed no histological abnormalities but a second biopsy two years later showed Marsh 3B and clinical and serological response to gluten-free diet. For 20 CD patients, no Marsh classification was available, but the CD diagnosis was based on the biopsies.

### Antibody assays and test procedures

IgA-aTTG and IgG-aDGL were measured (blinded to the histological diagnosis) in all sera with test kits from EUROIMMUN, Lübeck, Germany (cut-off ≥20 and ≥25 U/ml, respectively). IgA-EMA) were assessed in all except three sera by indirect immunofluorescence analysis using a combination of primate oesophagus, primate small intestine, and primate liver (EUROIMMUN).

We will argue in the results section below that a diagnostic procedure cannot simultaneously reach a PPV >90% and a NPV >90% for a broad range of prevalence without introducing a “grey zone”. We therefore define three possible outcomes for a diagnostic procedure: clearly negative, clearly positive and a grey zone containing the unclear cases. For a single antibody test this requires two cut-values.

Using the results of the individual antibody tests, we define two test procedures (algorithms). The first makes use of the IgA-aTTG test alone and the second combines it with the IgG-aDGL test.

We use the manufacturer's cut-off to define clearly negative cases and - following the suggestion of ESPGHAN - define a positive result using tenfold cut-off values. Values between these cut-values comprise the grey-zone.

**One-test-procedure**: CD if IgA-aTTG ≥10 ULN;

no CD if IgA-aTTG <1 ULN;

otherwise: unclear.

**Two-test-procedure**: CD if IgA-aTTG ≥10 ULN OR IgG-aDGL ≥10 ULN;

no CD if IgA-aTTG <1 ULN AND IgG-aDGL <1 ULN;

otherwise: unclear.

The diagnostic properties of the IgG-aDGL test alone will be discussed separately.

### Statistics

The three possible outcomes of the diagnostic procedure and two possible states of disease diagnosis lead to a 2 by 3 contingency table, Figure S2. Using it, we can estimate the diagnostic characteristics sensitivity and specificity, but also the proportion of false negative CD patients and false positive control patients (we refer to these as anti-sensitivity and anti-specificity, respectively). Note that they are not complements of sensitivity and specificity because of the indeterminate cases.

Assuming that sensitivity and specificity do not depend on prevalence, we can use Bayes' formula to determine the PPV and NPV and the proportion of indeterminate results, all as functions of prevalence. A detailed calculation is provided in the supplement.

We use maximum likelihood estimates for sensitivity, specificity, etc. Confidence bounds for PPV and NPV are determined based on a normal approximation for the logit-transformed quantity to avoid being overly optimistic [15].

Calculations and graphics were produced with R version 2.14.0 [16].

We consider a diagnostic procedure sufficiently reliable over a range of prevalence if two conditions are met: Firstly, that the PPV and NPV estimates both lie above 95%. Secondly, that the 95% lower confidence bound (LCB) for both predictive values be simultaneously above 90% over the whole range of prevalence. The second condition guaranties sufficient statistical precision for the first and can only be fulfilled with large enough data sets.

## Results

**Figure 1:**
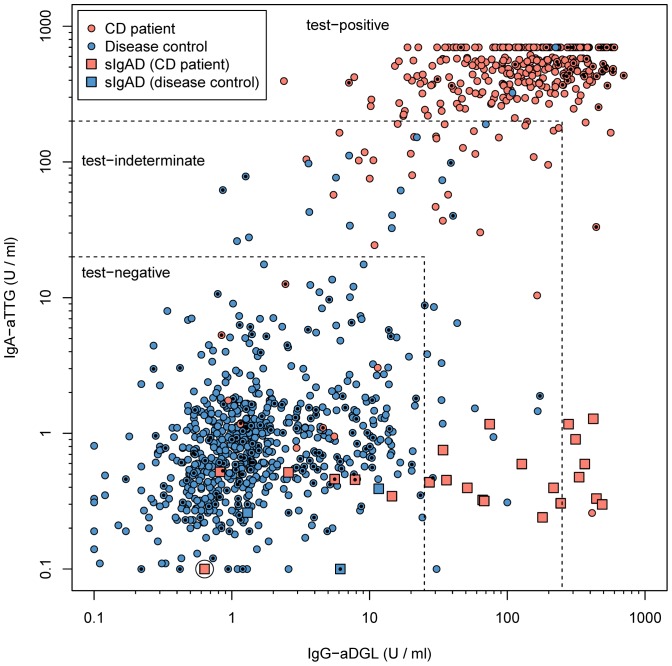
IgA-aTTG and IgG-aDGL in 376 CD patients (red) and 695 controls (blue). Patients with sIgAD are denoted by a larger square and a black dot in the centre indicates that the child was below two years of age. The large circle indicates that this is the CVID patient. Dashed lines show the three regions for the two-test procedure. Values smaller than 0.1 are depicted in the plot as though they were 0.1 and those greater than 700 as though they were 700. Note, that all patients with sIgAD had a concentration of IgA-aTTG below 2 U/ml.

The results of the antibody tests are presented in Figure 1. For a listing of clinical details regarding cases with discrepancies between diagnosis and antibody titres see Tables S1 and S2.

### Two-valued diagnostic procedures are insufficient

Many commercially available antibody tests only consider the outcome positive or negative. Applying company cut-offs for children without known sIgAD to our data (Table 1), specificity and sensitivity are 97.3% and 97.2% for IgA-aTTG, and 97.1% and 88.4% for IgG-aDGL. IgA-EMA is comparable to IgA-aTTG (97.0% and 96.8), but is more expensive and requires highly skilled staff. Despite high specificity and sensitivity, it turns out that PPV and NPV only meet the reliability criterion above (see statistics section above) for a narrow range of prevalence, if at all (Table 1).

**Table 1:** Performance of IgA-aTTG and IgG-aDGL assays at company cutoffs.

	Subjects without known sIgAD				All subjects			
	(352 CD and 692 control patients)				(376 CD and 695 control patients)			
	IgA-aTTG	IgG-aDGL	2 tests	≥1 test	IgA-aTTG	IgG-aDGL	2 tests	≥1 test
	≥20 U/ml	≥25 U/ml	≥cut-off	≥cut-off	≥20 U/ml	≥25 U/ml	≥cut-off	≥cut-off
True positives	342	311	309	344	342	329	309	362
True negative	673	672	686	659	676	675	689	662
False positives	19	20	6	33	19	20	6	33
False negatives	10	41	43	8	34	47	67	14
Sensitivity	0.972	0.884	0.878	0.977	0.909	0.875	0.821	0.963
Specificity	0.973	0.971	0.991	0.952	0.973	0.971	0.991	0.953
Prevalence range for reliable test	0.350–0.643	–	0.158–0.299	0.482–0.688	-	–	0.167–0.226	0.484–0.573

The prevalence range provides the interval for which the test procedure meets the reliability requirements of as defined in the statistics section. A dash indicates that no range exists for which these requirements are met. CD, coeliac disease; IgA-aTTG, IgA-antibodies to tissue transglutaminase; IgG-aDGL, IgG-antibodies to deamidated gliadin; sIgAD, selective IgA deficiency.

Figure 1 suggests that there are clearly negative cases with very low antibody concentrations and clearly positive cases with very high ones. In between there is a minority of unclear cases that spoil the predictive power of the antibody tests. This suggests the introduction of a third category “grey zone”, i.e. unclear test results

### Characteristics of the three valued diagnostic procedures

The three-valued procedures defined in the statistics section above do perform very well over a broad range of prevalence, because PPV and NPV are only calculated in cases in which the test procedures make a definite prediction.

Table 2 summarises performance in our data both after excluding patients with known sIgAD and for the entire cohort. Note that introducing the grey zone (diagnosis only possible after duodenal biopsy and evaluation of the histology) leads to a marked decrease in sensitivity as compared to using company cut-values because only clear results are considered. Nevertheless both PPV and NPV are nearly perfect (>98%) for the prevalence (34%) in our data.

**Table 2:** Comparison of the diagnostic procedures.

	Subjects without known sIgAD		All subjects	
	(352 CD and 692 control patients)		(376 CD and 695 control patients)	
	One-test-Procedure	Two-test-procedure	One-test-Procedure	Two-test-procedure
True positives	310	314	310	321
True negatives	673	659	676	662
False positives	2	2	2	2
False negatives	10	8	4	14
Number in grey zone	49	61	49	72
Sensitivity	0.881	0.892	0.824	0.854
Specificity	0.973	0.952	0.973	0.953
Anti-sensitivity	0.028	0.023	0.090	0.037
Anti-specificity	0.003	0.003	0.003	0.003
Prevalence range for reliable test	0.086–0.643	0.085–0.688	0.091–0.361	0.088–0.574
Proportion in grey zone	0.030–0.067	0.048–0.073	0.030–0.046	0.050–0.082

The prevalence range provides the interval for which the test procedure meets the reliability requirements of as defined in the statistics section. The proportion of children in the grey zone was calculated for the endpoints of the prevalence interval from the row above it. CD, coeliac disease; sIgAD, selective IgA deficiency.

**Figure 2:**
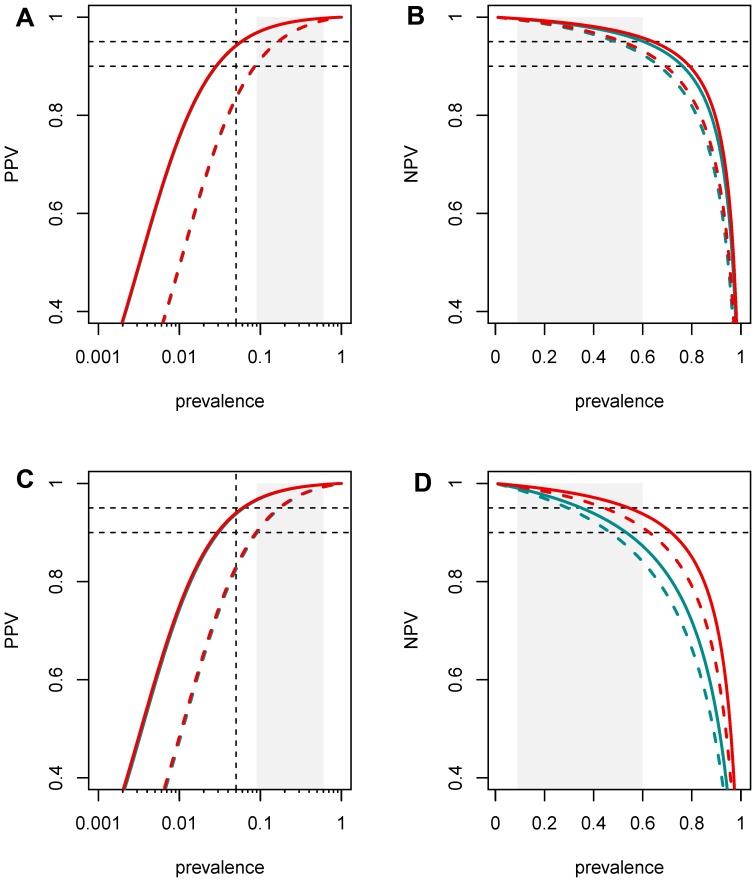
PPV and NPV (solid lines) plotted as functions of the prevalence together with a 95% lower confidence bound (LCB, dashed lines). The one-test procedure is shown in turquoise and the two-test procedure in red. The two procedures are virtually indistinguishable for PPV, but differ markedly for NPV when prevalence is high and all patients are included. The top two plots (**A** and **B**) are shown for the 1044 patients without known sIgAD and the bottom (**C** and **D**) two for all 1071 patients. Disease prevalence between 9% and 60% is shown by grey shading. PPV and NPV of better than 90% and 95%, respectively, are shown by dashed black lines as is the prevalence of 5%.

It is necessary to check the performance of the test procedures at much lower prevalence such as encountered in clinical settings: The dependence of predictive values on prevalence are shown in Figure 2 together with a 95% LCB. One can see that both test procedures fulfil the stringent criteria (specified in the statistics section above) for reliably diagnosing CD over the prevalence range from 9 to 64%. This also applies to the two-test-procedure when sIgAD patients are included, with the slight modification that the range becomes 9 to 57%. The one test-procedure is compromised by false negative cases, as expected (reliability criterion met only up to a prevalence of 36%).

As we see in Table 2, only about 5% of the children have an indeterminate test result. This rate varies from 3% at very low prevalence up to 11% with 100% prevalence.

Considering the IgG-aDGL test alone for all patients (positive ≥10 ULN, negative <1 ULN, otherwise unclear), one finds 106 true positives, no false positives, 675 true negatives, 47 false negatives and 243 unclear cases. Although this yields a PPV of 100%, the low number of true positives compared to the other procedures means that this estimate cannot be provided with a comparable certainty, i.e. the LCB is lower and one meets the stringent criteria only over a prevalence range of 19–29%.

### Patients under two years of age

**Figure 3:**
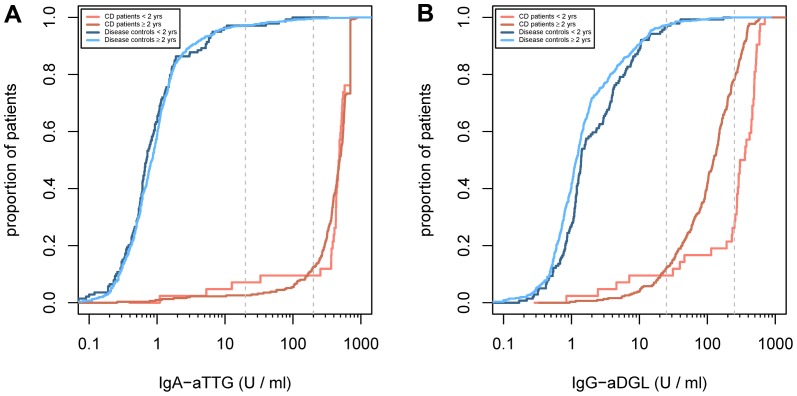
Empirical distribution functions IgA-aTTG and IgG-aDGL. in control patients (blue) and CD patients (red) comparing measurements in (**A**) children under two years (42 CD patients, 139 control patients) and (**B**) above (311 CD patients, 552 controls). Children with sIgAD were excluded. Vertical dashed lines show the two cut-values.

Some literature suggests that very young children show a delayed antibody response and thus antibody tests might have a compromised sensitivity [17], [18]. Plotting empirical cumulative distribution function of the antibody titres (Figure 3) in CD and control patients shows that the distribution in children under two years and above are comparable. If anything, IgG-aDGL response in very young CD patients appears to be stronger.

Thus, there is no evidence supporting an age restriction for the application of the diagnostic procedures discussed here. Since the conditional distributions of the antibody titres are super-imposable further subgroup analyses in age groups appear pointless and would lack power due to limited sample sizes.

### IgA-EMA in test-positive cases

ESPGHAN guidelines [2] require that a positive (≥10 ULN) IgA-aTTG test be confirmed by a positive IgA-EMA test in a different blood sample. Of the 312 subjects who were considered positive according to the one-test procedure, all (including two false-positives) were positive for IgA-EMA (data was unavailable for two subjects). Similarly, of the 323 subjects who were positive according to the two test procedure (among them two false-positives), only 9 were not positive for IgA-EMA. These 9 were all CD patients. Seven of them have sIgAD, one has IgA concentrations below age-specific cut-off, but above the level proposed for of sIgAD (0.07 g/l) [19]. The remaining case is a 1 year-old girl with CD and a histological finding of Marsh 3C.

### Antibodies in patients with inflammatory bowel disease

From 81 control patients with inflammatory bowel disease, 79 (one-test procedure) or 77 (two-test procedure) are correctly identified as negative and the remaining two/four are in the grey zone and would require a biopsy.

## Discussion

Until recently, the diagnosis of CD mainly relied on intestinal biopsies. New guidelines propose diagnosing CD in symptomatic patients without biopsy if the concentration of CD-specific antibodies exceeds 10 ULN and further confirmatory tests are positive [2].

In the future, the diagnostic strategy to exclude or confirm CD will mainly rely on CD-specific antibodies with intestinal biopsies relegated to difficult border-line cases. However, such diagnostic procedures must be shown to be reliable and in concordance with the earlier definition. This requires that PPV and NPV be simultaneously high over a broad range of prevalence.

For such a validation, large sample sizes for both CD and control patients are necessary. To the best of our knowledge our study including 376 CD patients and 695 controls is the largest study investigating antibody diagnostics in paediatric CD.

Simultaneously high PPV and NPV are very difficult to achieve unless one introduces a third diagnostic result “indeterminate” in addition to the conventional “positive” and “negative” test results. Calculating PPV and NPV only for those cases in which the diagnostic procedure has a definite result, i.e. excluding the grey zone cases, increases the predictive values.

Inspired by the ESPGHAN proposal [2] to regard IgA-aTTG values ≥10 ULN as diagnostic we consider two simple diagnostic procedures. The one-test procedure based on IgA-aTTG is positive for IgA-aTTG ≥10 ULN, negative if IgA-aTTG <1 ULN and indeterminate for values in between. Our two-test procedure combines two antibodies IgA-aTTG and IgG-aDGL: The diagnostic result is negative if both antibodies are <1 ULN, positive if (at least) one of them is ≥10 ULN and indeterminate otherwise.

We showed that if sIgAD is excluded both test procedures are reliable for a prevalence between 9 and 57%. Presumably, the range of prevalence is even broader, but more data would be needed to show this. The PPV for IgG-aDGL test alone with the ≥10 ULN is also very promising, but more studies are needed to verify these results [3].

Specialised gastroenterological clinics report very high prevalence of CD (39 to 92%) [20]–[23]. Given clinical suspicion of CD without prior antibody test [7]–[9] prevalence ranges between 3 to 10%. Down to this prevalence, the diagnostic procedures presented here yield reliable results. However, the prevalence in mass screening studies can be as low as 0.14 to 5.5% [24], [25].

Both the one and two-test procedure performed comparably well. Nevertheless we argue that the two-test procedure may be safer and advantageous in special situations. When total IgA measurements are not available (e.g. in very small children) measuring IgG-aDGL picks up IgA-deficient CD cases. Indeed, not excluding sIgAD patients from our data, the NPV of the one test procedure is compromised.

In addition, there are reports that the percentage of IgA-aTTG negative CD (without sIgAD) is as high as 8% [26] or even 24% [27]. We observed 10 (3%) of 352 such CD cases. In line with another report [20] about a quarter (2/10) were positive for IgG-aDGL when the company cut-off was applied, of which one had a partial IgA-deficiency (IgA below age specific reference range, but above 0.07 g/l) [19]. Note that the widespread routine of pre-screening with IgA-aTTG may lead to an underrepresentation of such patients in our data as well as other published data. Using the two-test procedure may help to counteract and safeguard against this selection bias.

Current guidelines [2] recommend confirming a positive (≥10 ULN) antibody test result with IgA-EMA positivity in a second blood sample. Looking at a second sample may be sensible to exclude a mix-up of samples. In our data, all 310 children positive for IgA-aTTG (≥10 ULN) were also IgA-EMA positive using the same sample. The PPV of both procedures is so high, that further confirmation by IgA-EMA or HLA-typing only adds negligible information. This strongly suggests that the purpose of a confirmatory IgA-EMA test is to exclude sample mix-up. For example, this may have shed new light on the two who were false positives. On the other hand, a negative result amongst those who are currently true positives, would only move them to the grey zone. The net result can only be an increasing (or unchanging) PPV without compromising NPV. We stress, however, that until confirming our results prospectively, EMA should maintain their diagnostic role as reported in the new ESPGHAN guidelines.

The diagnostic procedures also appear safe in children under two years of age. The point estimates of PPV and NPV for both diagnostic procedures are not markedly different from those in the entire population. We found no false positives among 139 control patients and three false negatives among 42 CD cases. Although some research suggests that one should be wary of negative antibody test results for children under the age of two, we see no indication in our data suggesting that the tests perform worse.

A few remarks on the limitations and properties of our data are in order. They were collected over a long period (15 years). Although the CD specific antibodies were measured centrally, we did not re-measure total IgA and thus had to rely on local results assessing sIgAD. We were not able to perform confirmatory assays on a second blood sample. Patients with sIgAD are enriched in our sample inflating differences between the one and the two-test-procedures when they are not excluded. Moreover, the method of acquiring data may well have led to an underrepresentation of unclear cases, both in terms of contradictory antibody results and uncertain diagnoses. When selecting our patients, we only had to exclude six cases with unclear diagnoses Figure S1). However, it is noteworthy we did not exclude the typically “difficult” situation of type I diabetes mellitus (23 children, 20 true positives, two true negatives, one unclear test result in either test procedure). Collecting details on the clinical symptoms that led to intestinal biopsy and details on the response to diet as well as a central review of the intestinal biopsy results were unfeasible.

To conclude, antibody assays could render biopsies unnecessary in the majority of children if experienced paediatric gastroenterologists evaluate the case, as recommended [2]. This suggestion only applies to the test kit used here and should be verified for the different assays on the market. The 10 ULN has different properties depending on the test kit and laboratory, highlighting the strong need for quality management in coeliac serology [28]. CD remains a clinical diagnosis, but the extent to which serology can assist in this diagnosis may be much higher than hitherto expected, though this still has to be assessed by prospective studies. As proposed by the ESPGHAN [2] and the American College of Gastroenterology [29], we are thus currently performing the prospective AbCD trial [10] to further confirm the results presented here and to provide further evidence.

## Supporting Information

Figure S1 **Selection of patients for current data analysis starting from the 1502 data sets with informed consent and antibody data.** Only six patients were excluded due to unclear diagnosis.
(DOCX)

Figure S2 **Calculation of PPV, NPV and proportion of patients without reliable diagnosis for given prevalence.** D- disease controls, D+ CD patients, T- test negative, T+ test positive, T? test result in grey zone, FN-false negative, FP-false negative, TN- true negative TP- true positive.
(DOCX)

Table S1 **Double positive (IgA-aTTG and IgG-aDGL) control patients.**
(DOCX)

Table S2 **IgA-aTTG negative CD patients.**
(DOCX)
