# Correction: Antibodies in the Diagnosis of Coeliac Disease: A Biopsy-Controlled, International, Multicentre Study of 376 Children with Coeliac Disease and 695 Controls

**DOI:** 10.1371/journal.pone.0105230

**Published:** 2014-08-01

**Authors:** The PLOS ONE Staff

There is an error in the third column of the “False negatives” row of Table 2. Please see the corrected Table 2 below.

**Table 2:** Comparison of the diagnostic procedures.

	Subjects without known sIgAD		All subjects	
	(352 CD and 692 control patients)		(376 CD and 695 control patients)	
	One-test-Procedure	Two-test-procedure	One-test-Procedure	Two-test-procedure
True positives	310	314	310	321
True negatives	673	659	676	662
False positives	2	2	2	2
False negatives	10	8	34	14
Number in grey zone	49	61	49	72
Sensitivity	0.881	0.892	0.824	0.854
Specificity	0.973	0.952	0.973	0.953
Anti-sensitivity	0.028	0.023	0.090	0.037
Anti-specificity	0.003	0.003	0.003	0.003
Prevalence range for reliable test	0.086-0.643	0.085-0.688	0.091-0.361	0.088-0.574
Proportion in grey zone	0.030-0.067	0.048-0.073	0.030-0.046	0.050-0.082

The prevalence range provides the interval for which the test procedure meets the reliability requirements of as defined in the statistics section. The proportion of children in the grey zone was calculated for the endpoints of the prevalence interval from the row above it. CD, coeliac disease; sIgAD, selective IgA deficiency

In addition, the sixth author’s name is incorrect. The correct name is Martin W. Laaß. The publisher apologizes for the error.
