# Supplementary material for: Antibodies in the Diagnosis of Coeliac Disease: A Biopsy-Controlled, International, Multicentre Study of 376 Children with Coeliac Disease and 695 Controls
Source: PLoS One. 2014 May 15;9(5):e97853. doi: 10.1371/journal.pone.0097853 (PMC4022637; doi:10.1371/journal.pone.0097853)
Supplement: Figure S1 — Selection of patients for current data analysis starting from the 1502 data sets with informed consent and antibody data. Only six patients were excluded due to unclear diagnosis. (DOCX) [file pone.0097853.s001.docx]

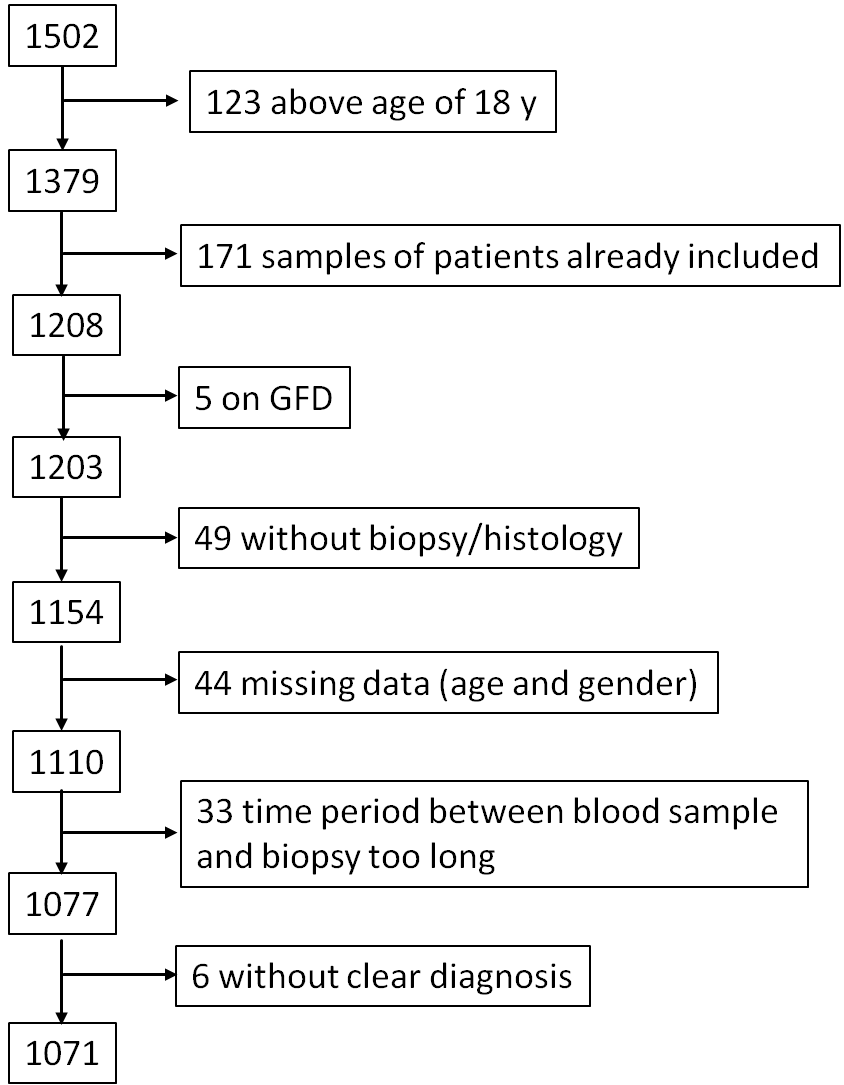


**Figure S1:** Selection of patients for current data analysis starting from the 1502 data sets with informed consent and antibody data. Only six patients were excluded due to unclear diagnosis.
