# Supplementary material for: Antibodies in the Diagnosis of Coeliac Disease: A Biopsy-Controlled, International, Multicentre Study of 376 Children with Coeliac Disease and 695 Controls
Source: PLoS One. 2014 May 15;9(5):e97853. doi: 10.1371/journal.pone.0097853 (PMC4022637; doi:10.1371/journal.pone.0097853)
Supplement: Figure S2 — Calculation of PPV, NPV and proportion of patients without reliable diagnosis for given prevalence. D- disease controls, D+ CD patients, T- test negative, T+ test positive, T? test result in grey zone, FN-false negative, FP-false negative, TN- true negative TP- true positive. (DOCX) [file pone.0097853.s002.docx]

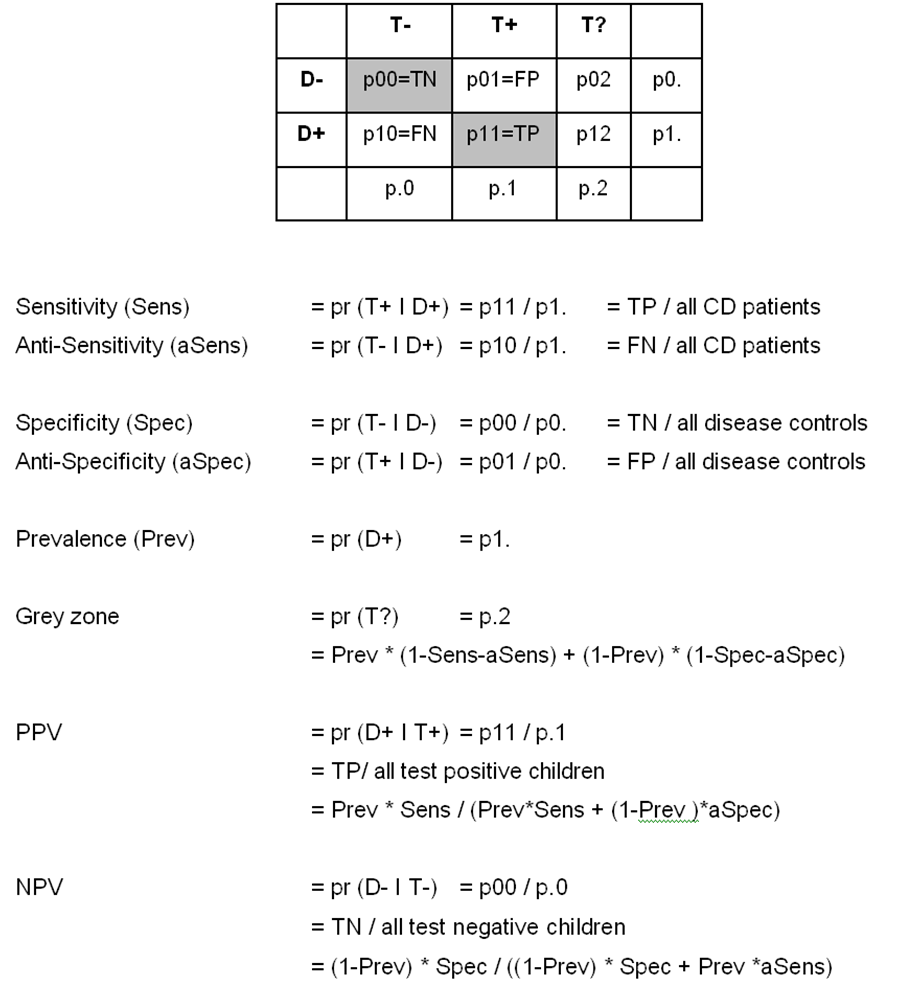


**Figure S2:** Calculation of PPV, NPV and proportion of patients without reliable diagnosis for given prevalence. D- disease controls, D+ CD patients, T- test negative, T+ test positive, T? test result in grey zone, FN-false negative, FP-false negative, TN- true negative TP- true positive.
