# Supplementary material for: Antibodies in the Diagnosis of Coeliac Disease: A Biopsy-Controlled, International, Multicentre Study of 376 Children with Coeliac Disease and 695 Controls
Source: PLoS One. 2014 May 15;9(5):e97853. doi: 10.1371/journal.pone.0097853 (PMC4022637; doi:10.1371/journal.pone.0097853)
Supplement: Table S1 — Double positive (IgA-aTTG and IgG-aDGL) control patients. (DOCX) [file pone.0097853.s003.docx]

**Table S1:** Double positive (IgA-aTTG and IgG-aDGL) control patients

| Patient | Age (years) | Gender | IgA-aTTG (U/ml) | IgG-aDGL (U/ml) | IgA-EMA  (Titre) | Compatible with Marsh grade | HLA | Diagnosis |
| --- | --- | --- | --- | --- | --- | --- | --- | --- |
| 1 | 1.8 | M | 40.1 | 40.4 | 1:100 | no | n.d. | Melena |
| 2 | 1.2 | M | 98.5 | 38.9 | negative | no | n.d. | Haematemesis |
| 3 | 8.8 | F | 73.4 | 33.7 | 1:32 | no | n.d. | Gastro-oesophageal reflux |
| 4* | 2.5 | M | 189.9 | 69.8 | 1:10 | no | DQ8+ | Type 1 diabetes mellitus |
| 5** | 3.5 | F | 270.0 | 99.1 | 1:100 | no | n.d. | Unclear, no CD |
| 6*** | 8.8 | F | ≥700.0 | 224.0 | 1:320 | Marsh 1 | n.d. | Cystic fibrosis |

IgA-aTTG, IgA-antibodies to tissue transglutaminase; IgG-aDGL, IgG-antibodies to deamidated gliadin; IgA-EmA, IgA-antibodies to endomysium; CD, coeliac disease; n.d., not determined

* Patient with abdominal pain, nausea, vomiting, and diarrhoea. Insufficient evidence for CD, but scheduled for re-biopsy.

** Patient is currently on a normal diet without any symptoms of CD. Before the biopsy, IgA-aTTG and IgA-EMA were performed locally With strongly positive results. Our test was performed on the same serum, but no further blood samples were taken. There were no histological signs of CD after inspection of seven biopsy specimens.

*** Two blood samples drawn at a later time were also both strongly positive for IgA-aTTG. A sampling error due to patchy lesions was suspected, but the patient could not be biopsied a second time.
