# Supplementary material for: Antibodies in the Diagnosis of Coeliac Disease: A Biopsy-Controlled, International, Multicentre Study of 376 Children with Coeliac Disease and 695 Controls
Source: PLoS One. 2014 May 15;9(5):e97853. doi: 10.1371/journal.pone.0097853 (PMC4022637; doi:10.1371/journal.pone.0097853)
Supplement: Table S2 — IgA-aTTG negative CD patients. (DOCX) [file pone.0097853.s004.docx]

**Table S2:** IgA-aTTG negative CD patients

| Age (years) | Gender | IgA-aTTG (U/ml) | IgG-aDGL (U/ml) | IgA-EMA (Titre) | IgA status |
| --- | --- | --- | --- | --- | --- |
| 1.0 | F | 1.10 | 4.59 | negative | normal |
| 1.1 | M | 0.46 | 7.84 | negative | sIgAD |
| 1.1 | M | 5.31 | 0.84 | negative | normal |
| 1.6 | F | 12.62 | 2.45 | negative | normal |
| 1.7 | M | 0.46 | 5.58 | negative | sIgAD |
| 2.4 | F | 0.78 | 2.95 | negative | normal |
| 3.0 | F | 0.52 | 0.82 | negative | sIgAD |
| 5.0 | M | 3.05 | 11.45 | negative | partial IgA deficiency* |
| 7.4 | M | 1.75 | 0.94 | 1:10 | normal |
| 8.9 | M | 0.00 | 0.63 | negative | CVID |
| 10.6 | M | 0.95 | 5.54 | negative | normal |
| 10.8 | F | 1.17 | 1.15 | negative | normal |
| 12.0 | M | 0.52 | 2.58 | negative | sIgAD |
| 15.0 | F | 0.34 | 14.53 | negative | sIgAD |
| 2.0 | F | 10.41 | 164.55 | 1:320 | normal |
| 2.3 | F | 0.30 | 488.70 | negative | sIgAD |
| 4.0 | F | 0.75 | 34.17 | negative | sIgAD |
| 4.0 | F | 0.32 | 67.98 | negative | sIgAD |
| 4.4 | F | 0.26 | 412.20 | negative | partial IgA deficiency |
| 5.0 | F | 0.45 | 36.14 | negative | sIgAD |
| 6.0 | M | 0.59 | 365.20 | negative | sIgAD |
| 6.0 | F | 0.59 | 127.42 | negative | sIgAD |
| 6.0 | F | 0.40 | 51.22 | negative | sIgAD |
| 8.0 | F | 0.32 | 66.22 | negative | sIgAD |
| 8.9 | M | 1.28 | 420.78 | negative | sIgAD |
| 9.5 | F | 0.33 | 443.85 | negative | sIgAD |
| 10.0 | F | 0.48 | 332.20 | negative | sIgAD |
| 10.0 | F | 1.17 | 278.60 | negative | sIgAD |
| 10.1 | F | 0.31 | 244.32 | negative | sIgAD |
| 12.0 | F | 0.44 | 27.25 | negative | sIgAD |
| 12.0 | F | 0.90 | 312.88 | negative | sIgAD |
| 16.0 | F | 0.24 | 180.05 | negative | sIgAD |
| 16.0 | F | 0.40 | 216.70 | negative | sIgAD |
| 16.0 | F | 1.17 | 74.53 | negative | sIgAD |

Patients above the solid line are not only negative for IgA-aTTG but also for IgG-aDGL (double-negative).

* Partial IgA deficiency (Yel et al. 2010): Total IgA < age specific reference range but > 0.07 g/l.
